# POLG-related disorders: Clinical and molecular Spectrum in the Saudi population

**DOI:** 10.1016/j.ymgmr.2026.101322

**Published:** 2026-05-25

**Authors:** Fuad Al Mutairi, Faisal Joueidi, Ziyad A. Al Mutairi, Maha Alshalan, Wafaa Eyaid, Aziza M. Mushiba, Malak AlGhamdi, Brahim Tabarki, Abdulaziz AlGhamdi, Ruqaiah AlTassan, Hamad Alzaidan, Abubakar Sharif, Hesham Aldhalaan, Muhammad Umair, Majid Alfadhel

**Affiliations:** a Genetic and Precision Medicine Department, King Abdullah Specialized Children Hospital, King Abdulaziz Medical City, Ministry of National Guard Health Affairs (MNG-HA), Riyadh, Saudi Arabia; b King Abdullah International Medical Research Center (KAIMRC), King Saud bin Abdulaziz University for Health Sciences, Ministry of National Guard Health Affairs (MNG-HA), Riyadh, Saudi Arabia; c College of Medicine, Alfaisal University, Riyadh, Saudi Arabia; d College of Medicine, King Saud Bin Abdulaziz University for Health Sciences, Ministry of National Guard Health Affairs (MNG-HA), Riyadh, Saudi Arabia; e Section of Medical Genetics, Children's Specialist Hospital, King Fahad Medical City, Riyadh, Saudi Arabia; f Medical Genetics Division, Department of Pediatrics, College of Medicine, King Saud University, King Saud University Medical City, Riyadh, Saudi Arabia; g Pediatric Neurology, Department of Pediatrics, Prince Sultan Military Medical City, Riyadh, Saudi Arabia; h Department of Medical Genomics, Genomic Medicine Centre of Excellence, King Faisal Specialist Hospital, and Research Centre, Riyadh, Saudi Arabia; i Organ Transplant Center of Excellence, King Faisal Specialist Hospital and Research Centre, Riyadh, Saudi Arabia; j Pediatric Neurology, King Faisal Specialist Hospital and Research Center, Riyadh, Saudi Arabia.; k Medical Genomics Research Department, King Abdullah International Medical Research Center (KAIMRC), King Saud Bin, Abdulaziz University for Health Sciences, Ministry of National Guard Health Affairs (MNGH), Riyadh, Saudi Arabia

**Keywords:** POLG, Myopathy, Mitochondrial replication, Mitochondrial depletion, Developmental delay, Seizures

## Abstract

**Background:**

POLG-related disorders are a group of mitochondrial diseases caused by variants in the *POLG* gene, which is essential for mitochondrial DNA replication and repair. These disorders encompass a wide spectrum of clinical manifestations, ranging from severe, early-onset conditions to milder, adult-onset syndromes.

**Methods:**

We conducted a retrospective study of 19 molecularly confirmed cases with POLG-related disorders from 16 unrelated families in six different referral centers. Clinical, radiological, and molecular analysis were performed following standard methods.

**Results:**

Most of the patients in this study presented with variable neurological symptoms before the age of 12 years (80%); commonly, these symptoms included developmental delay and encephalopathy (63%), seizures (58%), ataxia and dysphagia (42% each). Molecular analysis revealed eight different disease-causing variants in the *POLG* gene. The most frequently observed variant was c.3286C > T; p.(Arg1096Cys). Notably, the *POLG* c.1957G > A; p.(Glu653Lys) variant has not been reported in the literature previously, and might impact protein folding and stability.

**Conclusion:**

Despite the management of these conditions remaining largely supportive, advances in understanding the molecular mechanisms of POLG-related disorders offer promise for future therapeutic strategies targeting mitochondrial function and stability. This study highlights the complexity of POLG-related disorders and underscores the need for continued research into their pathophysiology and treatment.

## 1 Background

Nuclear-encoded proteins play a crucial role in mitochondrial DNA biogenesis. The replication of mitochondrial DNA relies heavily on DNA polymerase gamma (POLG), an enzyme encoded by the nuclear *POLG* gene. This interdependence highlights the complex relationship between nuclear and mitochondrial genomes in cellular function [1]. The human *POLG* gene, which comprises 23 exons, was characterized and cloned in 1996. Later, in 2001, researchers identified the first *POLG* variant linked to a human disease, specifically progressive external ophthalmoplegia (PEO) [2]. The underlying mechanisms of mitochondrial DNA (mtDNA) variant can be attributed to two primary sources: spontaneous errors during DNA replication or unrelated damage that introduces miscoding lesions. These factors are responsible for most base substitution variants in mtDNA. Furthermore, these processes are likely linked to the gradual accumulation of point variants and deletions in mtDNA as organisms age at the cellular level [3]. POLG demonstrates remarkably accurate DNA replication with very few nucleotide incorporation errors. This exceptional fidelity is due to its high nucleotide selectivity and exonucleolytic proofreading activity. Combined, these characteristics enable POLG to replicate DNA with minimal errors, making it a crucial component in maintaining genetic integrity**.** [4].

Several factors contribute to the development of POLG-related disorders, including genetic factors (mtDNA or nuclear DNA), immune dysfunction, and environmental factors such as mitochondrial toxins and viral infections [3]. The *POLG* variants are associated with a wide spectrum of phenotypes, depending on age of onset and electron transport activity, and two patterns of inheritance have been attributed to autosomal dominant and recessive modes, with an estimated prevalence of 0.3 per 100,000 adults [5]. In POLG-related disorders, the spectrum of clinical manifestations has variable onset and severity, varying between childhood to adulthood-onset and dominant or recessive inheritance, with manifestations including progressive external ophthalmoplegia, myoclonic epilepsy, sensory ataxia, intractable seizures, psychomotor regression and hepatic impairment [3], [6], [7]. The complexity of the genotype-phenotype overlapping POLG-related disorders is still unclear. This makes it challenging to categorize patients with an onset from early life to late adulthood using clinical findings and other factors. Thus, it is difficult to predict disease progression and to identify effective management strategies [1], [3].

Patients with POLG-related disorders exhibit a broad and overlapping clinical spectrum between the phenotypic presentations that were historically separated, including mitochondrial DNA depletion syndrome 4 A (Alpers type), mitochondrial DNA depletion syndrome 4B (MNGIE type), mitochondrial recessive ataxia syndromes (including SANDO and SCAE), and progressive external ophthalmoplegia (PEO) with either autosomal dominant or autosomal recessive inheritance [7], [8]. Childhood-onset mitochondrial DNA depletion syndromes are classically grouped into three main categories: Alpers–Huttenlocher syndrome (AHS), the myocerebrohepatopathy spectrum (MCHS), and myoclonic epilepsy. Importantly, clinical and biochemical variability in these disorders cannot be reliably predicted from POLG genotype, polymerase-γ phenotype, or measured enzymatic activity [3], [9]. The diagnosis of POLG-related disorders is established by a multidisciplinary evaluation. There are no specific blood or urine biomarkers; however, peripheral blood levels of lactate and plasma alanine can be evaluated. Muscle biopsies from affected individuals with POLG-related disorders may exhibit typical mitochondrial abnormalities, including fibers lacking cytochrome *c* oxidase activity, ragged-red fibers, or excessive lipid accumulation. These histological findings are characteristic markers of mitochondrial dysfunction in affected muscle tissues [9], [10]. MRI results can be normal in many patients with POLG-related disorders, however, this does not rule out the presence of the condition. While the MRI may appear unremarkable, especially in the early stages of the disease, it can sometimes reveal abnormalities in the bilateral nigrostriatal dopaminergic region and patchy gray matter in the occipital lobes, particularly in the striate cortex, basal ganglia, and thalamus [11]. Patients with AHS usually experience rhythmic high-amplitude delta with superimposed poly spikes, while the electroencephalogram (EEG) may be non-specifically abnormal in POLG-related disorders; it is typically observed in the occipital lobe predilection [12]. Although over a hundred *POLG* variants have been reported in the literature, additional disease-causing variants continue to be identified, making their pathogenicity difficult to determine. Molecular genetic testing is a useful way to identify the causative variants in most cases [3], [7]. Recent decades have seen significant progress in diagnosing mitochondrial diseases. Mitochondria-targeted gene therapies have been successfully reported in animal models for some disorders and are a potential therapeutic approach for POLG-related disorders. However, effective treatments that can modify the course of these diseases remain elusive [13]. Promising therapies, such as deoxynucleosides and eoxycytidine/deoxythymidine combination therapy, are undergoing trials in POLG-related disorders. Their true efficacy can only be determined through rigorous, randomized controlled trials. These studies are crucial for establishing the effectiveness of treatments for conditions associated with *POLG* gene variants [14], [15], [16].

In this cohort, we report the clinical, biochemical, and mutation spectrum of POLG-related disorders in Saudi Arabia. This retrospective analysis aimed to provide a comprehensive understanding of the natural history of these disorders in the region.

## 2 Materials and methods

### 2.1 Patients and data collection

This study retrospectively analyzed medical records of twenty patients diagnosed with POLG-related disorders at six tertiary referral centers in Riyadh, Saudi Arabia, from January 2015 to December 2024. POLG-related disorders were included based on established criteria, encompassing neurological presentations and confirmed through DNA molecular genetic testing. The following data were collected from each patient: age, sex, age of onset and diagnosis, presence of myopathy, neuroregression, global developmental delay, history and type of seizures, and motor and cognitive skills assessed at variable times. There are currently no established standardized clinical diagnostic criteria for POLG-related disorders. Diagnosis is based on recognition of characteristic clinical features in combination with molecular confirmation of pathogenic *POLG* variants, supported by laboratory, neuroimaging, and ophthalmic findings when available. Supporting laboratory testing and brain MRI findings were included when available. The diagnoses of POLG-related disorders were ultimately confirmed through molecular testing, either by Sanger or next-generation sequencing (NGS), to detect pathogenic, likely pathogenic, and validated recessive variants in the *POLG* gene. Data are presented using summary statistics due to the small sample size and proportion of missing data (Supp- 1).

### 2.2 Genetic testing

The diagnosis of POLG-related disorders in this cohort was confirmed by whole exome sequencing (WES) or whole genome sequencing (WGS) of genomic DNA for most patients. For a small number of patients with previously confirmed cases in the family, diagnosis was made according to relevant gene panels or single-gene or Sanger testing by accredited commercial laboratories. The variants were classified according to the guidelines of the ACMG [17]. A minor allele frequency threshold of <0.001 was applied based on our internal database (1410 exomes), including 13,180 WES/WGS performed at different CAP-accredited laboratories, as well as the Genome Aggregation Database (gnomAD), and the Exome Aggregation Consortium (ExAC). Additionally, the variants were fully segregated within available family members. Variants obtained after WES/WGS were analyzed and filtered using standard methods. Additionally, all variants identified via WES/WGS were validated by bidirectional Sanger sequencing following standard methods.

### 2.3 3D Structural modeling of POLG

The human POLG protein sequence was retrieved from the UniProt database and used to model the mutant variant (E653K) using MODELLER. Additionally, the same sequence was submitted to the I-TASSER server for ab initio and template-based structure prediction. The resulting models were evaluated based on confidence scores to determine the reliability and structural quality of the predicted conformations.

## 3 Results

### 3.1 Clinical findings

Nineteen patients (12 males and 7 females) diagnosed with POLG-related disorders were included from 16 unrelated families (Supp-1). 10 out of 19 cases resulted in mortality (52%). The progression of the illness led to steady deterioration over time, ultimately resulting in death in some patients, with an average survival period of five years from the onset of the symptoms. Consanguinity was reported in all cases except three families (84%), and positive family history in 10 cases (53%). Most of the patient symptoms began during the first few years of life, typically before the age of twelve (16 cases, 80%). One patient presented clinically in the neonatal period with lactic acidosis and a sepsis-like presentation, which required aggressive supportive care. Coincidentally, this patient was also diagnosed with Tyrosinemia type 1 and responded to the Nitisinone (NTBC) treatment protocol. The eldest case in this cohort was a 45-year-old who complained of a mild form of myopathy at 14 years of age and was diagnosed based on family history segregation as a heterozygous. The most common presenting signs were developmental delay and, encephalopathy (12 cases each, 63%), seizures (11 cases, 58%), ataxia and dysphagia (nine cases, 42% each), myopathy (seven cases, 37%), and dysarthria and cerebellar findings (three cases, 16%) (Table 1). Visual impairment was noticed in 47% of cases. Symptoms such as progressive ophthalmoplegia (PEO) and optic atrophy were seen in 25% and 20% of cases, respectively. Finally, respiratory symptoms and insufficiency were seen in two cases (10%). From a laboratory standpoint, persistent lactic acidosis was observed in 9 cases (47%), whereas Creatine phosphokinase (CPK) elevations were infrequently reported. Elevated liver enzymes (LFTs), with and without cholestasis, were observed in four patients (20%). Radiological findings from brain MRI scans revealed white matter disease in four cases (20%). The management of POLG-related disorder epilepsy was extremely challenging in most of these cases. The seizures were refractory, necessitating the use of multiple antiepileptic medications (AEMs), including levetiracetam, phenobarbital, carbamazepine, and lacosamide. Additionally, a mitochondrial drug cocktail, consisting of biotin, l-carnitine, and Coenzyme Q10, was employed as adjunctive therapy in five cases.

**Table 1:** Summary of the clinical symptoms associated with POLG-related disorders reported in this study and in the literature.

Clinical Findings	This study	Jha et al. 2022	Hikmat et al. 2020	R. Horvath et al. 2006	Wong et al. 2008	Sha Tang et al. 2011
Developmental delay	(12/19; 63%)	(14/22; 63.7%)	–	–	(40/61; 65%)	–
Ataxia	(9/19; 47%)	–	(87/138; 63%)	(10/38; 26%)	(∼10/61; 16%)	(15/73; 20%)
Myopathy	(8/19; 42%)	(10/22; 45%)	(68/135 (50%)	(16/38; 42%)	(22/61; 36%)	(36/73; 49%)
Encephalopathy	(12/19; 63%)	(11/22; 50%)	(79/101; 78%)	(15/38; 40%)	–	–
Epilepsy	(11/19; 58%)	(11/22; 50%)	(107/155; 69%)	(7/38; 20%)	(33/61; 54%)	(36/73; 49%)
Ophthalmoplegia	(5/19; 25%)	(8/22; 36.4%)	(56/146; 38%)	(19/38; 50%)	(12/61; 20%)	(24/73; 33%)
Optic atrophy	(4/19; 20%)	(3/22;13.6%)	(55/146; 38%)	–	–	(7/73; 10%)
Liver dysfunction	(4/19; 20%)	(8/22; 36.4%)	(95/151; 64%)	(10/38; 26%)	(23/61; 38%)	(19/73; 26%)
Lactic acidosis	(9/19; 47%)	(6/17; 35%)	(29/84; 35%)	–	(19/61; 31%)	(22/73; 30%)
Survival Rate	(9/19; 47%)	–	(61/151; 39%)	40% (AHS, MCHS)		

### 3.2 Molecular study data

A total of seven different *POLG* variants were identified in the 19 individuals described in this study (Table 1). The majority of cases (17 cases, 90%) revealed homozygous variants consistent with an autosomal recessive inheritance pattern, while only two cases revealed heterozygous variants. The most frequently observed variant was the common pathogenic homozygous variant c.3286C > T; p.(Arg1096Cys), accounting for 11 cases (58%). The variants c.1156C > T; p.(Arg386Cys) and c.925C > T; p.(Arg309Cys) were each identified in two cases. Four other variants, c.911 T > G; p.(Leu304Arg), c.2419C > T; p.(Arg807Cys), c.2620 T > A; p.(Leu874Met), and c.1957G > A; p.(Glu653Lys) were each reported in one case. All variants identified in this cohort are classified as pathogenic or likely pathogenic, except for c.1957G > A; p.(Glu653Lys) and c.2620 T > A; p.(Leu874Met), which are considered variants of uncertain significance (VUS). The *POLG* c.1957G > A variant predicted to result in the amino acid substitution p.Glu653Lys. To our knowledge, this variant has not been reported in the literature. This variant has a minor allele frequency of 0.00089% in individuals of European (non-Finnish) descent in gnomAD.

### 3.3 Structural impact of the POLG E653K mutation

**Fig. 1:**
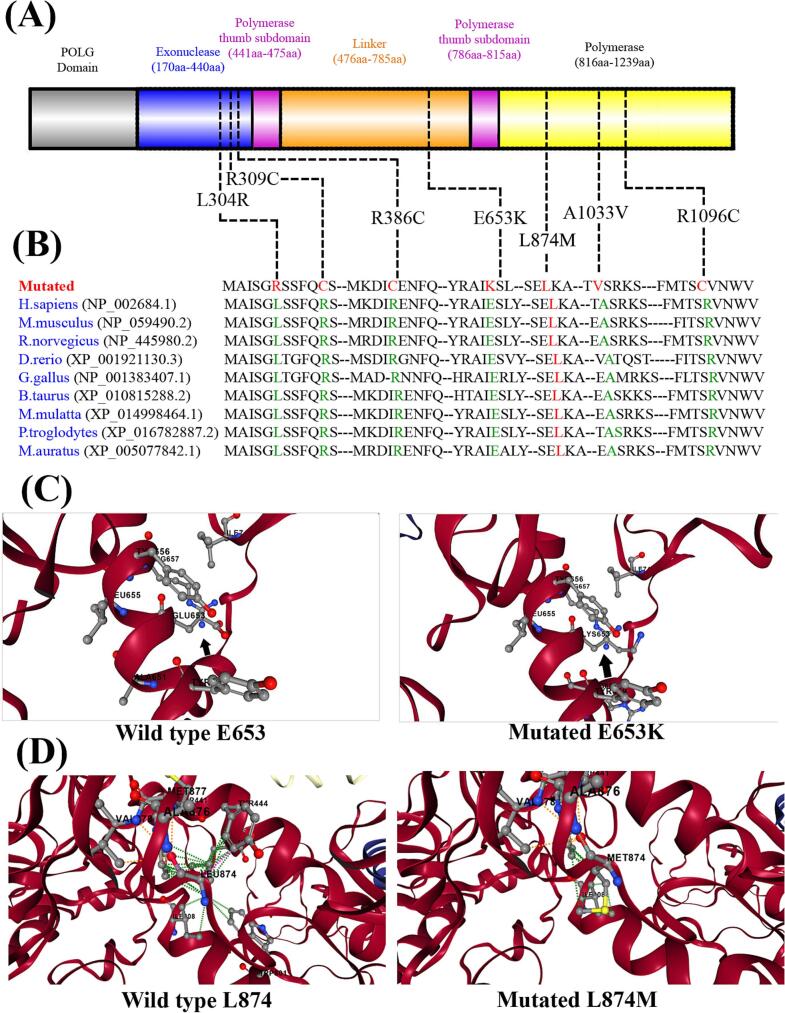
Structural and conservation analysis of POLG variants. (A) Schematic representation of the human POLG protein, highlighting the functional domains and the locations of variants identified in this study. Key domains include the Exonuclease domain (170–440 aa), Polymerase thumb subdomain I (441–475 aa), Linker region (476–785 aa), Polymerase thumb subdomain II (786–815 aa), and the Polymerase domain (816–1239 aa). (B) Partial amino acid sequence alignment of POLG across multiple species, demonstrating high evolutionary conservation of the mutations identified in this study, suggesting their potential functional significance. (C) Comparative 3D structural modeling of the wild-type and E653K-mutant POLG protein. The E653K substitution is predicted to disrupt secondary structure and may alter the protein's overall conformation or interfere with critical intramolecular interactions, potentially impacting POLG's polymerase function. (D) 3D modeling of the wild-type and Leu874Met mutant POLG protein suggests that the Leu874Met substitution may disrupt local secondary structure, potentially altering the overall conformation and functional integrity of the protein.

A structural comparison between the wild-type and mutant POLG protein highlights the effects of the E653K substitution. In the native protein, glutamic acid (Glu653) is a negatively charged residue, likely participating in salt bridges or hydrogen bonds that contribute to local structural stability. The variant replaces Glu653 with lysine (Lys), a positively charged residue, resulting in a reversal of local electrostatic charge. This alteration disrupts the native electrostatic environment and may interfere with critical intramolecular interactions. The predicted change in folding free energy (ΔΔG = −0.63 kcal/mol) indicates a slight destabilization of the protein structure. Although this energy difference is modest, it could still impact protein folding, stability, or polymerase function, particularly if residue 653 lies within or near a catalytic domain or an interaction interface (Fig. 1).

## 4 Discussion

This study presents the largest cohort of POLG-related disorders in Saudi Arabia to date, with most cases recruited from referral centers in Riyadh. The disease's phenotype spectrum ranges from early-onset neonatal metabolic acidosis to late-onset neurological symptoms, including ataxia and myopathic manifestations. Typical features of early-onset POLG-related disorders (before age 12) include liver dysfunction, feeding difficulties, seizures, hypotonia, and muscle weakness [18]. At present, only ten patients from the original group have survived. The main cause of death is primarily related to CNS manifestations, which can lead to swallowing difficulties and dysphagia. These issues often result in recurrent chest infections, ultimately leading to cardiopulmonary arrest (Suppl Fig. A). None of the cases experienced mortality associated with status epilepticus; however, encephalopathy and refractory seizures were additional risk factors linked to a poor prognosis. The results of this research support the perspective that infantile cases presenting with lactic acidosis and hepatic symptoms are linked to the earliest disease manifestations and are associated with early mortality. Additionally, follow-up data for survival analysis from previous reports [20], [21] revealed a strong association between the age of disease onset and patient survival duration; individuals with earlier onset MCHS and AHS phenotypes exhibited poorer outcomes and a 39–40% mortality rate (Supplementary Fig-A). Moreover, further investigation demonstrated that the presence of neurological symptoms was significantly linked to a less favorable prognosis.

Gastrointestinal symptoms, specifically swallowing difficulties and dysphagia, were observed in 10 patients (50%), similar to previously published prevalence, and were usually associated with additional severe neurological phenotypes. Less commonly reported gastrointestinal symptoms in both adults and children include vomiting, delayed stomach emptying (gastroparesis), and chronic intestinal pseudo-obstruction (CIPO). Liver involvement was identified in 50–64% of the patients with POLG-related disorders, regardless of the age of onset [9], [19]. This study also demonstrated liver involvement in only four cases. For instance, in case #12, the patient experienced rapid liver deterioration in the neonatal period and was found to have a homozygous pathogenic variant in the *FAH* gene, confirming the diagnosis of autosomal recessive tyrosinemia type I. This patient was still alive at 10 months of age. Moreover, another three cases showed mild cholestasis and elevated LFTs, and two of these patients were still alive.

Due to the nature of childhood motor development, symptoms such as hypotonia, developmental delay, and feeding difficulties are likely to manifest early in the course of the disease for most cases. However, the onset of other symptoms, including seizures, peripheral neuropathy, ataxia, and muscle weakness, varies considerably among affected individuals [20]. We noticed 55% of children in this cohort experienced seizures as the initial or progressing symptom. These seizures manifested in various forms, including focal seizures, generalized seizures, myoclonic seizures, and status epilepticus. Among these, Epilepsia Partialis Continua seizures were noted in two patients (Supp-1). This is similar to the previously reported diverse range of seizure presentations, which highlights the complexity of AHS and underscores the importance of early recognition and diagnosis [8], [9], [21]. Most of the patients received different AEMs along with mitochondrial cocktails, which showed variable responses regardless of their genotypes. Visual impairments leading to blindness may appear months to years after the onset of other neurologic manifestations. Brain MRI scans conducted on some patients with POLG-related disorders in this cohort revealed a spectrum of abnormalities. These findings ranged from hyperintense signal irregularities in the right cortical and subcortical white matter areas to pronounced atrophy and volume reduction. Notably, these abnormal neuroimaging results correlated with unfavorable prognoses and reduced survival rates among the affected individuals. Furthermore, our study revealed that commonly used laboratory tests in the initial diagnostic evaluation of mitochondrial disorders, such as CPK, lactate, routine acylcarnitine profiles, and urine organic acid analysis, did not consistently align with the genotype or mortalities.

A total of seven different *POLG* variants were identified in this study. The majority of patients with early-onset disease (before the age of 12) had homozygous pathogenic *POLG* variants (regardless of the variant types, but mostly c.3286C > T; p.(Arg1096Cys). Individuals harboring heterozygous *POLG* variants had a better prognosis compared to those with homozygous variants. The common pathogenic homozygous variant c. 3286C > T; p.(Arg1096Cys) was associated with seizures and a low survival rate in all affected cases (Supplementary Fig. 1). In this cohort, two cases with the variant c. 1156C > T; p.(Arg386Cys) are still alive without significant neurological or hepatic manifestations. The evidence supporting the pathogenicity of the *POLG* variant c.1957G > A (p.Glu653Lys) includes several clinical and laboratory findings. The patient was diagnosed by WES after presenting with metabolic myopathy and mildly elevated levels of CPK, along with a positive family history. Furthermore, the abnormal EMG study suggests myopathy characterized by muscle membrane irritability, while the muscle biopsy revealed focal myopathic features and uneven staining for NADH and SDH. Additionally, the ultrastructural examination showed focal subsarcolemmal mitochondrial accumulation, indicating mitochondrial myopathies. We performed comparative 3D structural modeling of the wild-type POLG protein and the E653K mutant, which is predicted to disrupt the protein's secondary structure, potentially altering its overall conformation and interfering with critical intramolecular interactions, which may impact POLG's polymerase function (Fig. 1).

While all the patients included in this study were of Saudi Arabian descent, the retrospective design resulted in difficulties in acquiring certain clinical data, but it did highlight the clinical spectrum of POLG-related disorders in one ethnicity. Future systematic prospective studies should yield more comprehensive and reliable data to enhance our understanding of this devastating disorder and provide further guidance for targeted therapy.

The following are the supplementary data related to this article.

Supplementary Fig. 1 (A) Figure shows the percentages of the symptoms in this study compared to previous publications. (B) Figure shows the mortality rate related to the symptoms in this study. (C) Figure shows the mutation spectrum in this study and mortalities related to the genotype. (D) Figure shows the symptoms related to the genotype in this study.

Supplementary Table 1

## Details of ethics approval

All procedures followed were in accordance with the ethical standards of the committee responsible for human experimentation (institutional and national) and with the Helsinki Declaration of 1975. This study was approved by King Abdullah International Medical Research Center IRB (IRB Number: NRR24/116/12).

## CRediT authorship contribution statement

**Fuad Al Mutairi:** Writing – review & editing, Writing – original draft, Visualization, Validation, Project administration, Methodology, Formal analysis, Data curation, Conceptualization. **Faisal Joueidi:** Formal analysis, Data curation. **Ziyad A. Al Mutairi:** Formal analysis, Data curation. **Maha Alshalan:** Data curation. **Wafaa Eyaid:** Formal analysis, Data curation. **Aziza M. Mushiba:** Formal analysis, Data curation. **Malak AlGhamdi:** Formal analysis, Data curation. **Brahim Tabarki:** Formal analysis, Data curation. **Abdulaziz AlGhamdi:** Formal analysis, Data curation. **Ruqaiah AlTassan:** Formal analysis, Data curation. **Hamad Alzaidan:** Formal analysis, Data curation. **Abubakar Sharif:** Formal analysis, Data curation. **Hesham Aldhalaan:** Formal analysis, Data curation. **Muhammad Umair:** Writing – review & editing, Validation, Software, Methodology, Formal analysis, Data curation. **Majid Alfadhel:** Writing – review & editing, Validation, Formal analysis, Data curation.

## Informed consent

The parents of the participating patients gave informed consent for the publication of the case details.

## Funding

This study received no specific funding from any financial support agency, whether from the public, commercial, or not-for-profit sector.

## Declaration of competing interest

The authors declare that they have no conflict of interest.

## Data Availability

Data will be made available on request.
