# Supplementary figures and images for: POLG-related disorders: Clinical and molecular Spectrum in the Saudi population
Source: Mol Genet Metab Rep. 2026 May 25;47:101322. doi: 10.1016/j.ymgmr.2026.101322 (PMC13226778; doi:10.1016/j.ymgmr.2026.101322)

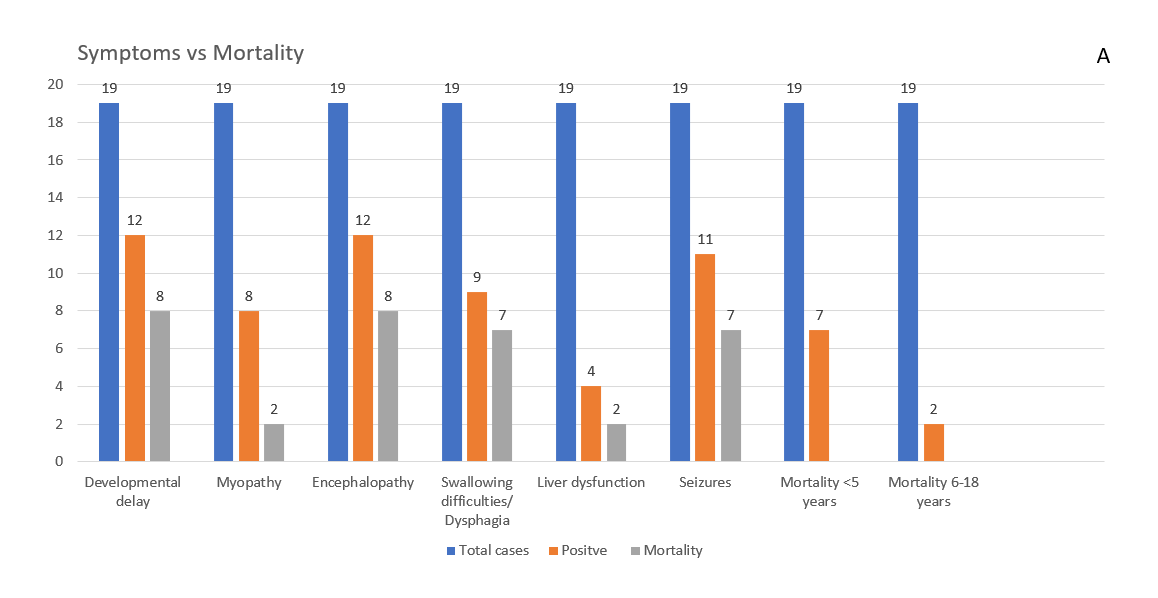


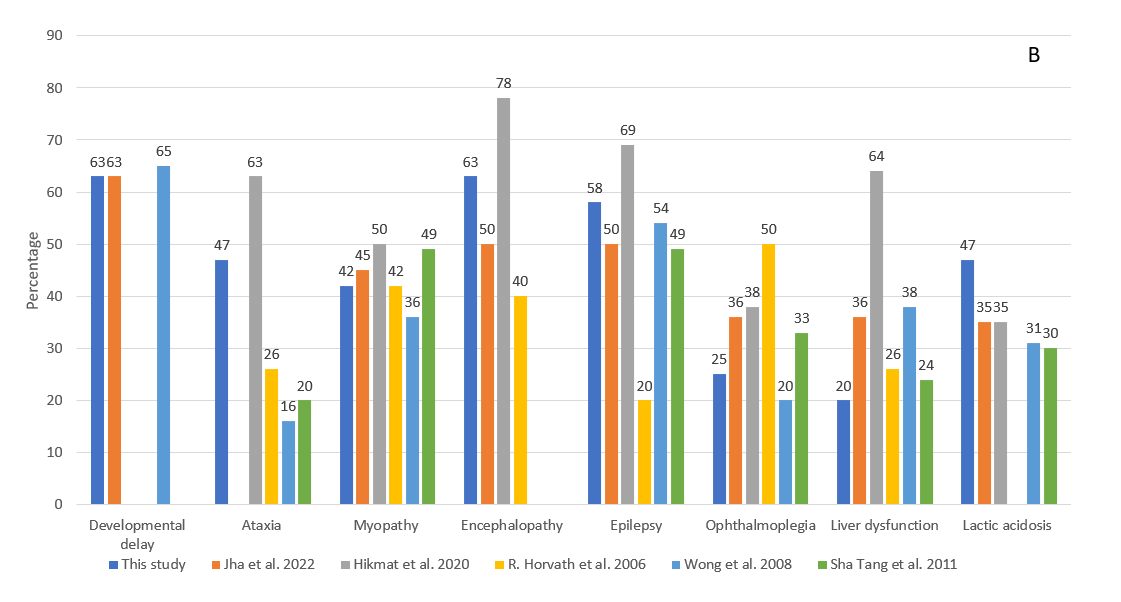

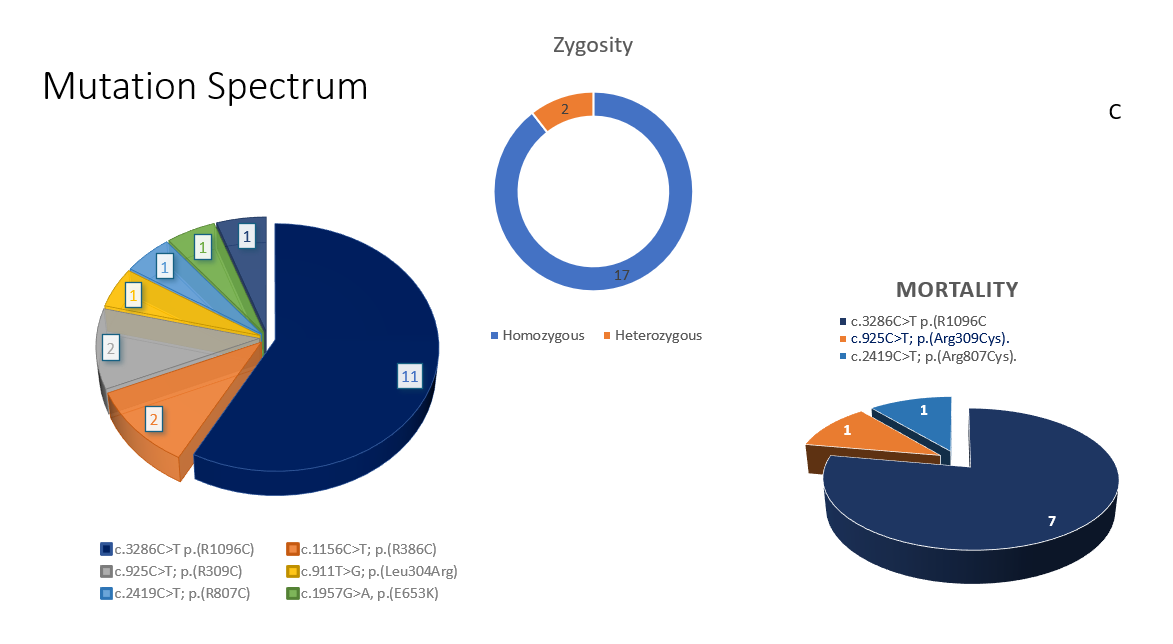


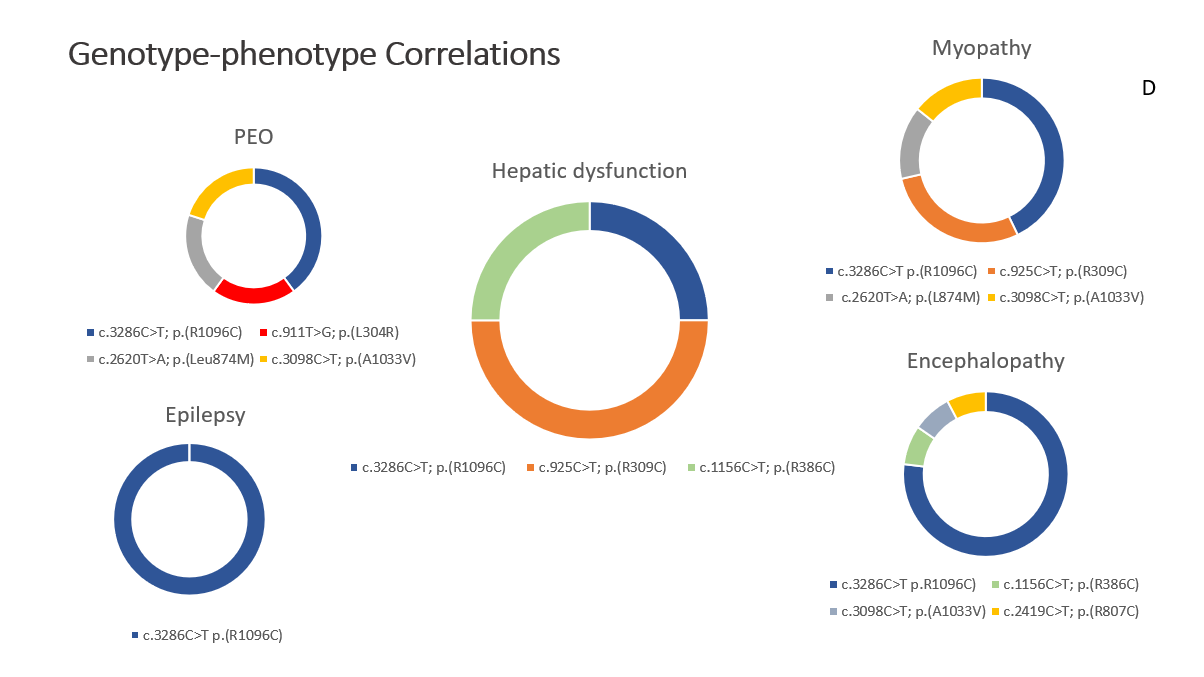

Supplement: Supplementary Fig. 1 — (A) Figure shows the percentages of the symptoms in this study compared to previous publications. (B) Figure shows the mortality rate related to the symptoms in this study. (C) Figure shows the mutation spectrum in this study and mortalities related to the genotype. (D) Figure shows the symptoms related to the genotype in this study. [file mmc1.docx]
