# Supplementary material for: POLG-related disorders: Clinical and molecular Spectrum in the Saudi population
Source: Mol Genet Metab Rep. 2026 May 25;47:101322. doi: 10.1016/j.ymgmr.2026.101322 (PMC13226778; doi:10.1016/j.ymgmr.2026.101322)
Supplement: Supplementary Table 1 [file mmc2.docx]

**Table S-1:** Clinical description of the patients identified in the present study.

|  | **Gender** | **Presentation age** | **Respiratory insufficiency** | **Myopathy** | **PEO** | **Visual Impairment** | **Optic atrophy** | **Developmental delay** | **Ataxia** | **Dysarthria / cerebellar findings** | **Seizures** | **Swallowing difficulties/ Dysphagia** | **Family History** | **Consanguinity** | **Cholestasis/ transaminitis** | **Lactic acidosis** | **CPK** | **White matter abnormalities** | **Treatment Provided** | **Died\ Alive** | **Variant / Zygosity** |
| --- | --- | --- | --- | --- | --- | --- | --- | --- | --- | --- | --- | --- | --- | --- | --- | --- | --- | --- | --- | --- | --- |
| 1 | Male | 1.5 y | No | No | No | Yes | Yes | Yes, severe | No | No | Yes, Epileptic encephalopathy | Yes | No | Yes | Normal | High | Normal | No | Levetiracetam, Coenzyme Q-10, L-Carnitine | Died | c.3286C>T; p.(Arg1096Cys). Pathogenic, Homozygous |
| 2 | Female | 2 y | No | No | No | Yes | Yes | Yes, severe | No | No | Yes | Yes | No | Yes | Abnormal | High | Normal | Not Done | Levetiracetam | Died | c.3286C>T; p.(Arg1096Cys). Pathogenic, Homozygous |
| 3 | Female | 1 y | No | No | No | Yes | Yes | Yes, severe | No | No | Yes, Epilepsia Partialis Continua | Yes | No | Yes | Normal | High | Normal | No | Levetiracetam, Phenobarbital, Carbamazepine, Lacosamide | Died | c.3286C>T; p.(Arg1096Cys). Pathogenic, Homozygous |
| 4 | Male | 4 y | No | No | No | Yes | No | Yes | Yes | No | Yes, Epilepsia Partialis Continua | No | Yes | No | Normal | Normal | Normal | No | None | Died | c.3286C>T; p.(Arg1096Cys). Pathogenic, Homozygous |
| 5 | Male | 1.5 y | No | No | No | No | No | Yes | Yes | UK | Yes | No | No | Yes | Normal | Normal | Normal | Yes | Phenobarbital, Topiramate, Pyridoxine | Died | c.3286C>T; p.(Arg1096Cys). Pathogenic, Homozygous |
| 6 | Male | 3 y | Yes | No | Yes | Yes | Yes | Yes | UK | UK | Yes | Yes | Yes. | Yes | Normal | High | Normal | Yes | Levetiracetam, Clobazam, Carbamazepine, Pyridoxine, Riboflavin, L-Carnitine, Thiamine, Biotin, Coenzyme Q-10 | Died | c.3286C>T; p.(Arg1096Cys). Pathogenic, Homozygous |
| 7 | Male | 7 y | Yes | Yes | Yes | No | No | Yes | Yes | Yes | Yes | Yes | Yes | Yes | Normal | Normal | Not Done | Yes | Levetiracetam, Clobazam, Riboflavin, L-Carnitine, Thiamine, Biotin, Pyridoxine, Coenzyme Q-10, Lipoic acid | Died | c.3286C>T; p.(Arg1096Cys). Pathogenic, Homozygous |
| 8 | 6 years/ M | 6 m | No | No | No | No | No | Yes, severe | No | No | Yes, Epileptic encephalopathy, LGS, recurrent status | Yes | Yes | Yes | Normal | Normal | Not Done | No | Levetiracetam, Phenobarbital, Topiramate, vigabatrin tried with poor compliance | Alive | c.3286C>T; p.(Arg1096Cys). Pathogenic, Homozygous |
| 9 | 5 years / F | 1.5 years | No | Yes | No | No | No | Yes | Yes | No | Yes | No | No | No | Normal | Normal | Not done | No | Levetiracetam | Alive | c.3286C>T; p.(Arg1096Cys). Pathogenic, Homozygous |
| 10 | 18 years/ F | 15 y | No | Yes | Yes | Yes, bilateral ptosis | No | No | Yes | Yes | Yes | No | Yes | Yes | Normal | Normal | Not Done | Not Done | Levodopa, Levetiracetam, Phenobarbital, Topiramate | Alive | c.3286C>T; p.(Arg1096Cys). Pathogenic, Homozygous |
| 11 | 12 years/ F | 5 y | No | No | No | No | No | No | No | No | Yes | No | Yes | Yes | Normal | Normal | Not Done | Not Done | Phenobarbital, Levetiracetam & Topiramate | Alive | c.3286C>T; p.(Arg1096Cys). Pathogenic, Homozygous |
| 13 | 14 years/ M | 2 y | No | No | No | No | No | Yes, severe | Yes | Yes | No | No | No | Yes | Normal | High | Normal | Yes | None | Alive | c.1156C>T; p.(Arg386Cys). Pathogenic, Homozygous |
| 14 | 10 months/ M | 1 m | No | No | No | No | No | No | UK | UK | No | No | Yes (Tyrosinemia type 1) | Yes | Abnormal | High | Normal | Not Done | NTBC for the tyrosinemia 1 | Alive | c.1156C>T; p.(Arg386Cys). Pathogenic, Homozygous |
| 15 | Male | 9 y | No | Yes | No | Yes, bilateral ptosis | No | No | Yes | No | No | Yes | Yes | Yes | Abnormal | High | Normal | No | Coenzyme Q-10, L-Carnitine | Died | c.925C>T; p.(Arg309Cys). Pathogenic, Homozygous |
| 16 | 18 years/ F | 13 y | No | Yes | No | Yes, bilateral ptosis | No | No | Yes | No | No | Yes | Yes | Yes | Abnormal | Normal | High | No | Coenzyme Q-10 | Alive | c.925C>T; p.(Arg309Cys). Pathogenic, Homozygous |
| 12 | 12 years/ M | 5 y | No | Yes | Yes | No | No | No | Yes | No | No | No | Yes | Yes | Normal | High | Normal | No | UK | Alive | c.911T>G; p.(Leu304Arg). Pathogenic, Homozygous |
| 17 | 45 years / M | 14 y | No | Yes | Yes | Yes | No | No | No | No | No | No | No | Yes | Normal | Normal | High | No | None | Alive | c.2620T>A; p.(Leu874Met). VUS, Heterozygous |
| 18 | Female | 9 m | No | No | No | No | No | Yes, severe | UK | No | No | Yes | Yes | Yes | Normal | High | Normal | No | UK | Died | c.2419C>T; p.(Arg807Cys). Pathogenic, Homozygous |
| 19 | 27 years/ M | 21 y | No | Yes | No | No | No | No | No | No | No | No | Yes | No | Normal | Normal | High | Not Done | None | Alive | c.1957G>A; p.(Glu653Lys). VUS, Heterozygous |
